# Adjuvant transcatheter arterial chemoembolization after curative resection for hepatocellular carcinoma patients with solitary tumor and microvascular invasion: a randomized clinical trial of efficacy and safety

**DOI:** 10.1186/s40880-018-0331-y

**Published:** 2018-10-10

**Authors:** Wei Wei, Pei-En Jian, Shao-Hua Li, Zhi-Xing Guo, Yong-Fa Zhang, Yi-Hong Ling, Xiao-Jun Lin, Li Xu, Ming Shi, Lie Zheng, Min-Shan Chen, Rong-Ping Guo

**Affiliations:** 1 State Key Laboratory of Oncology in South China, Collaborative Innovation Center for Cancer Medicine, Sun Yat-sen University Cancer Center, Guangzhou, 510060 Guangdong P. R. China; 2 Department of Hepatobiliary and Pancreatic Surgery, Sun Yat-sen University Cancer Center, Guangzhou, 510060 Guangdong P. R. China; 3 Department of Pathology, Sun Yat-sen University Cancer Center, Guangzhou, 510060 Guangdong P. R. China; 4 Department of Medical Imaging, Sun Yat-sen University Cancer Center, Guangzhou, 510060 Guangdong P. R. China

**Keywords:** Solitary tumor, Hepatocellular carcinoma, Adjuvant therapy, Transcatheter arterial chemoembolization, Hepatectomy alone, Microvascular invasion

## Abstract

**Background:**

The optimal strategy for adjuvant therapy after curative resection for hepatocellular carcinoma (HCC) patients with solitary tumor and microvascular invasion (MVI) is controversial. This trial evaluated the efficacy and safety of adjuvant transcatheter arterial chemoembolization (TACE) after hepatectomy versus hepatectomy alone in HCC patients with a solitary tumor ≥ 5 cm and MVI.

**Methods:**

In this randomized, open-labeled, phase III trial, HCC patients with a solitary tumor ≥ 5 cm and MVI were randomly assigned (1:1) to receive either 1–2 cycles of adjuvant TACE after hepatectomy (Hepatectomy-TACE) or hepatectomy alone (Hepatectomy Alone). The primary endpoint was disease-free survival (DFS); the secondary endpoints included overall survival (OS) and adverse events.

**Results:**

Between June 1, 2009, and December 31, 2012, 250 patients were enrolled and randomly assigned to the Hepatectomy-TACE group (*n *= 125) or the Hepatectomy Alone group (*n *= 125). Clinicopathological characteristics were balanced between the two groups. The median follow-up time from randomization was 37.5 months [interquartile range 18.3–48.2 months]. The median DFS was significantly longer in the Hepatectomy-TACE group than in the Hepatectomy Alone group [17.45 months (95% confidence interval [CI] 11.99–29.14) vs. 9.27 months (95% CI 6.05–13.70), hazard ratio [HR] = 0.70 (95% CI 0.52–0.95), *P *= 0.020], respectively. The median OS was also significantly longer in the Hepatectomy-TACE group than in the Hepatectomy Alone group [44.29 months (95% CI 25.99–62.58) vs. 22.37 months (95% CI 10.84–33.91), HR = 0.68 (95% CI 0.48–0.97), *P* = 0.029]. Treatment-related adverse events were more frequently observed in the Hepatectomy-TACE group, although these were generally mild and manageable. The most common grade 3 or 4 adverse events in both groups were neutropenia and liver dysfunction.

**Conclusion:**

Hepatectomy followed by adjuvant TACE is an appropriate option after radical resection in HCC patients with solitary tumor ≥ 5 cm and MVI, with acceptable toxicity.

## Introduction

Hepatocellular carcinoma (HCC) is the sixth most common malignancy worldwide [1] and the second leading cause of cancer-related death in China [2]. An estimated 466,100 new HCC cases and 422,100 deaths occurred in China in 2015 [3]. Surgical resection remains the main radical treatment for HCC, although the recurrence rate after hepatectomy is high and hampers further improvement in the prognosis of HCC patients [4, 5]. The conventional risk factors for recurrence include tumor size, multiple lesions, vascular invasion, poor differentiation, and tumor rupture [6–8]. Over the past decade, microvascular invasion (MVI) has been proposed as a potential risk factor for recurrence after hepatectomy [9, 10]. Recent studies have confirmed the significance of MVI in postoperative recurrence [11–13]. A previous study by our research group also showed that the recurrence rate was over 50% for HCC patients with solitary tumor ≥ 5 cm and MVI, where MVI was confirmed as the only independent risk factor for overall survival (OS) and disease-free survival (DFS) among that cohort [14].

Different therapeutic agents and/or approaches have been evaluated as adjuvant therapy for HCC after curative resection, including interferon [15], oral chemotherapeutic agents (1-hexylcarbamoyl-5-fluorouracil (HCFU) [16] and capecitabine [17]), hepatic arterial infusion chemotherapy [18], and targeted therapy (sorafenib) [19]. Unfortunately, it has been shown that most of these approaches did not reduce the risk of recurrence or were poorly tolerated, and, most importantly, these strategies were not associated with significant survival benefits [20]. As such, an optimal adjuvant therapy with respect to efficacy, safety, and cost-effectiveness remains to be defined. Our previous phase III randomized clinical study indicated that transcatheter arterial chemoembolization (TACE) may be an appropriate adjuvant therapy option for stage IIIA HCC patients [21]. Therefore, this present phase III clinical trial was designed to evaluate the efficacy and safety of radical hepatectomy plus adjuvant TACE (Hepatectomy-TACE), compared with radical hepatectomy alone (Hepatectomy Alone), in HCC patients with solitary tumor ≥ 5 cm and MVI after curative resection.

## Methods

### Trial design

This study was an open-labeled, randomized, phase III trial conducted at the Sun Yat-sen University Cancer Center (Guangzhou, China), designed to evaluate the efficacy and safety of radical hepatectomy plus adjuvant TACE versus radical hepatectomy alone among HCC patients with solitary tumor ≥ 5 cm and MVI after curative resection. The protocol and all modifications were approved by the Institutional Review Board and Ethics Committee of our cancer center. This study complied with the Declaration of Helsinki and the Good Clinical Practice Guidelines (International Conference on Harmonisation of Technical Requirements for Registration of Pharmaceuticals for Human Use (ICH), Version E6) [22]. All patients provided written informed consents. This study was registered in ClinicalTrials.gov (http://ClinicalTrials.gov, trial number NCT02788526) on March 23, 2016.

### Eligibility criteria

The eligibility criteria for inclusion were as follows: 18–75 years of age; histologically confirmed HCC with MVI (MVI was defined by the presence of tumor emboli within either the central hepatic vein, the portal, or the large capsular vessels [23]); Eastern Cooperative Oncology Group performance score (ECOG PS) ≤ 2; no previous treatment for HCC; solitary tumor ≥ 5 cm before surgery confirmed by 2 radiological examinations (ultrasonography with computer tomography or magnetic resonance imaging); R0 resection; no evidence of recurrence at radiological follow-up at 3–5 weeks after surgery; adequate hematologic, hepatic, and renal functions. The exclusion criteria included histologically positive resection margin (R1 resection); evidence of recurrence at radiological follow-up 3–5 weeks after surgery; history of organ transplantation; active uncontrolled infection; allergy to any TACE agent; other malignancies over the preceding 5 years before the HCC diagnosis, except for adequately treated carcinoma in situ of the cervix and squamous or basal cell carcinoma of the skin; pregnancy, breastfeeding, or lack of use of adequate contraception among women of childbearing potential; neurological or psychiatric disorders that may affect cognitive assessment and inform consent; concomitant antitumor therapy or participation in other interventional clinical trials.

### Hepatectomy

All surgical resection procedures were performed following the techniques described in our previous study [10]. Briefly, routine abdominal exploration was carefully performed to evaluate the extent of the tumor and to exclude extrahepatic metastases. After adequate mobilization of the liver, we used intraoperative ultrasound (ALOKA SSD-5500, Tokyo, Japan) to assess the number of lesions and tumor size, the presence of MVI, and the extent of resection. During tumor removal, the liver parenchyma was separated using the Cavitron Ultrasonic Surgical Aspirator (Integra LifeSciences CUSA Excel, Plainsboro, NJ, USA), and the involved vessels were ligated. The Pringle maneuver was also applied to occlude blood inflow to the liver.

### Randomization

All patients were screened for enrollment at the first follow-up (3–5 weeks after hepatectomy). Full patient assessment, including demographic characteristics, medical history, physical examination, routine blood analysis (hematology and biochemistry), and radiological examinations [computed tomography (CT) or magnetic resonance imaging (MRI)], were performed within 1 week of the study enrollment. The patients with evidence of recurrence during the screening for enrollment were excluded. Then the eligible patients were randomly assigned (at a 1:1 ratio) to receive either 1–2 cycles of adjuvant TACE (Hepatectomy-TACE group) or routine follow-up without adjuvant treatment (Hepatectomy Alone group). Randomization was performed using a sealed envelope system according to a predesigned random number.

### Adjuvant TACE

The patients in the Hepatectomy-TACE group underwent TACE 4–6 weeks after hepatectomy according to liver function and performance status. TACE was performed using the techniques we have described previously [24]. In brief, a catheter was placed into the proper hepatic artery through the femoral artery using the Seldinger technique, hepatic arterial angiography was performed, and 200 mg/m^2^ carboplatin (Carboplatin, Bristol-Myers Squibb, New York, NY, USA) and 6 mg/m^2^ mitomycin (Mitomycin, Hisun, Taizhou, China) were infused followed by 4–5 mL of the emulsion of iodized oil (lipiodol, Andre Guerbet, Aulnay-sous-Bois, France) and 40 mg/m^2^ epirubicin (Epirubicin Hydrochloride, ‎Pfizer, New York, NY, USA). After 4–6 weeks, these patients underwent a complete assessment consisting of physical examination, routine blood analysis, and CT scan. The second cycle of TACE was performed according to the decision of investigators based on the patients’ conditions and the assessment results.

### Follow-up

All patients were followed-up at an interval of 2–3 months. To avoid the potential effect of hepatitis B virus (HBV) reactivation on recurrence, all patients with positive serum hepatitis B surface antigen (HBsAg) were administered with routine antiviral therapy with lamivudine (GlaxoSmithKline, Brentford, UK; 100 mg, once daily) or entecavir (Bristol-Myers Squibb, New York, USA; 0.5 mg, once daily). At each follow-up visit, physical examination, blood test (serum alpha-fetoprotein [AFP] and liver function), and enhanced abdominal CT or MRI scan were performed. Once suspicious recurrence/metastasis was detected, further examinations including hepatic angiography or biopsy were conducted. Recurrence/metastasis was confirmed based on the cytologic/histologic evidence or on the non-invasive diagnostic criteria for HCC by the European Association for the Study of Liver [7]. Patients with recurrence in both groups received subsequent treatment according to the decision of the multi-disciplinary team of our cancer center. Adverse events (AEs) were recorded from the day of randomization to the last day of follow-up. Toxicity was evaluated according to the National Cancer Institute Common Terminology Criteria for Adverse Events (version 3.0). The study was censored on March 31, 2016.

### Statistical analyses

The primary endpoint was DFS and was defined as from the time of randomization to the diagnosis of recurrence or death from any cause. The secondary endpoints included OS, which was defined as from the time of randomization to the date of the last follow-up or death, and AEs.

Assuming an increase in median DFS of 6 months between the Hepatectomy-TACE group (18.0 months) and Hepatectomy Alone group (12.0 months) [hazard ratio (HR) 0.66], it was estimated that 176 events and a total of 210 patients (105 in each group) were required for randomization to achieve a statistical power of 85% with a significance level of 0.05 for a one-sided error. All analyses were performed according to the per-protocol principle. Survival curves were estimated using the Kaplan–Meier method and compared using the log-rank test. The median survival with 95% confidence interval (CI) was calculated. Cox proportional analyses were performed to estimate HRs with 95% CIs. The *t*-test was used for group comparisons of AEs. We also performed subgroup analyses for sex (male vs. female), age (< 60 years vs. ≥ 60 years), ECOG PS (0 vs. 1–2), tumor size (5–10 cm vs. > 10 cm), cirrhosis (present vs. absent), and resection margin (< 2 cm vs. ≥ 2 cm). All statistical tests were performed with the Statistical Package for the Social Sciences (SPSS) (version 23, Chicago, IL, USA), Stata (version 13, College Station, TX, USA), and Medcalc (version 16.1, Acacialaan, Belgium) statistical software, and *P* values < 0.05 were considered significant.

## Results

### Patient characteristics and treatment administration

**Fig. 1:**
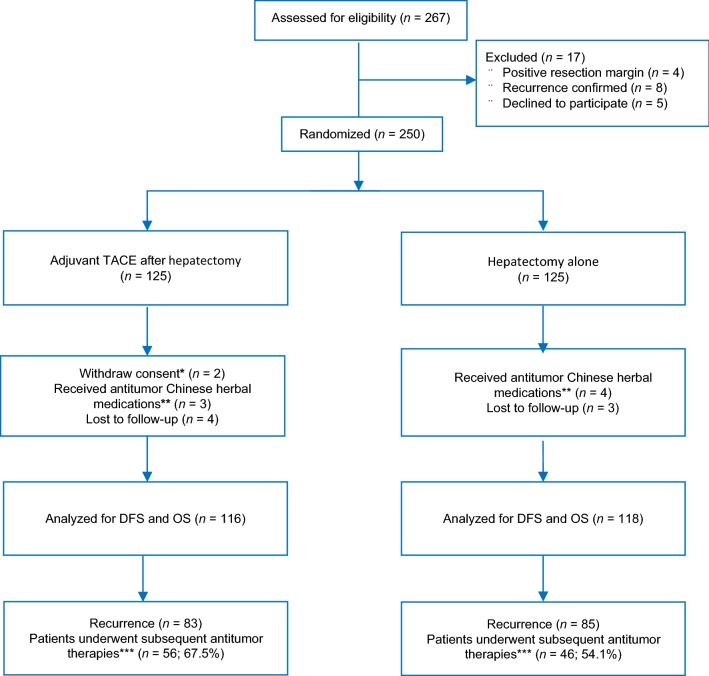
A flow diagram illustrating the overall patient enrollment, randomization, and outcomes of this study

Between June 1, 2009, and December 31, 2012, 250 patients were enrolled and randomly assigned to receive 1–2 cycles of adjuvant TACE after radical hepatectomy (the Hepatectomy-TACE group, *n* = 125) or hepatectomy alone (the Hepatectomy Alone group, *n* = 125). In the Hepatectomy-TACE group, 2 patients withdrew consent because of the potential toxicity of TACE, 3 patients had antitumor Chinese herbal prescriptions with HCC indications, and 4 patients were lost to follow-up. These patients were therefore excluded from the analysis. In the Hepatectomy Alone group, 4 patients had antitumor Chinese herbal prescriptions, and 3 patients were lost to follow-up and were also excluded from the analysis (Fig. 1).

Baseline demographic and clinical characteristics were well balanced between the two groups (Table 1). The median follow-up time for the entire cohort was 37.5 months from randomization [interquartile range (IQR), 18.3–48.2 months]. In the Hepatectomy-TACE group, 55 patients underwent 1 cycle of TACE, and 61 patients underwent 2 cycles of TACE.

**Table 1:** Baseline characteristics of the investigated hepatocellular carcinoma patients

Characteristic	Hepatectomy-TACE group (*n* = 116)	Hepatectomy Alone group (*n* = 118)	*P*-value
Age [years; median (range)]	44.0 (18–75)	48.5 (18–74)	0.112
Gender [cases (%)]			
Male	106 (91.4)	106 (89.8)	0.824
Female	10 (8.6)	12 (10.2)	
ECOG performance status [cases (%)]			
0	48 (41.4)	53 (44.9)	0.794
1	65 (56.0)	63 (53.4)	
2	3 (2.6)	2 (1.7)	
Serum HBsAg [cases (%)]			
Positive	94 (81.0)	101 (85.6)	0.384
Negative	22 (19.0)	17 (14.4)	
Preoperative serum AFP [cases (%)]			
< 25 ng/mL	37 (31.9)	36 (30.5)	0.888
≥ 25 ng/mL	79 (68.1)	82 (69.5)	
Hemoglobin (g/L; mean ± SD)	142.3 ± 23.7	141.2 ± 24.4	0.711
Platelet (× 10^9^/L; mean ± SD)	205.2 ± 79.1	178.6 ± 80.0	0.119
Alanine aminotransferase (U/L; mean ± SD)	55.3 ± 57.4	45.1 ± 27.9	0.086
Serum albumin (g/L; mean ± SD)	41.4 ± 5.7	41.6 ± 3.4	0.741
Serum total bilirubin (mol/L; mean ± SD)	14.4 ± 5.8	17.3 ± 24.6	0.212
Prothrombin time (s; mean ± SD)	12.2 ± 1.2	12.2 ± 1.1	0.725
Child–Pugh grade [cases (%)]			
Class A	116 (100.0)	116 (98.3)	0.498
Class B	0 (0.0)	2 (1.7)	
Serum urea (mmol/L; mean ± SD)	5.0 ± 1.6	5.2 ± 1.3	0.378
Serum creatinine (μmol/L; mean ± SD)	78.1 ± 24.4	73.8 ± 15.5	0.114

Continuous variables were compared by Student t-test; categorical variables are compared by the χ^2^ test or the Fisher’s exact test

Hepatectomy-TACE: radical hepatectomy followed by adjuvant transcatheter arterial chemoembolization; Hepatectomy Alone: had undergone only radical hepatectomy; HBsAg: hepatitis B surface antigen; AFP: alpha-fetal protein; SD: standard deviation

### Operative variables and postoperative outcomes

The operative variables and postoperative outcomes observed from the first day after hepatectomy till the date of discharge are summarized in Table 2. Twenty-four and 23 complications occurred in the Hepatectomy-TACE and Hepatectomy Alone group, respectively, including grade 1–2 fever, ascites, transient jaundice, pleural effusion, and hypoalbuminemia. One patient in the Hepatectomy-TACE group and 2 patients in the Hepatectomy Alone group experienced grade 3 liver bleeding. No patient died of complications during hospitalization.

**Table 2:** Operative variables and postoperative outcomes of the enrolled patients upon undergoing radical hepatectomy

Characteristic	Hepatectomy-TACE group (*n *= 116)	Hepatectomy alone group (*n* = 118)	*P*-value
Cirrhosis [cases (%)]			
Present	50 (43.1)	42 (35.6)	0.285
Absent	66 (56.9)	76 (64.4)	
Tumor size [cases (%)]			
5–10 cm	82 (70.7)	97 (82.2)	0.055
> 10 cm	34 (29.3)	21 (17.8)	
Operation time (min)	173.2 ± 48.6	182.3 ± 65.2	0.225
Operation blood loss (mL)	518.9 ± 441.6	421.6 ± 353.3	0.064
Blood transfusion [cases (%)]	26 (22.4)	18 (15.3)	0.183
Extent of liver resection [cases (%)]			
Major	45 (38.8)	46 (39.0)	1.000
Minor	71 (61.2)	72 (61.0)	
Resection margin [cases (%)]			
< 2 cm	91 (78.4)	92 (78.0)	1.000
≥ 2 cm	25 (21.6)	26 (22.0)	
Postoperative complications [cases (%)]	24 (20.7)	23 (19.5)	0.871
Grade 1			
Fever	5 (4.3)	3 (2.5)	0.497
Grade 2			
Fever	2 (1.7)	1 (0.8)	0.620
Ascites	5 (4.3)	3 (2.5)	0.497
Transient jaundice	5 (4.3)	6 (5.1)	1.000
Pleural effusion	3 (2.6)	3 (2.5)	1.000
Hypoalbuminemia	3 (2.6)	5 (4.2)	0.722
Grade 3			
Liver bleeding	1 (0.9)	2 (1.7)	1.000

Continuous variables are compared by Student *t*-test; categorical variables are compared by the χ^2^ test or the Fisher’s exact test

Hepatectomy-TACE: radical hepatectomy followed by adjuvant transcatheter arterial chemoembolization; Hepatectomy Alone: had undergone only radical hepatectomy

### Efficacy of treatment

**Fig. 2:**
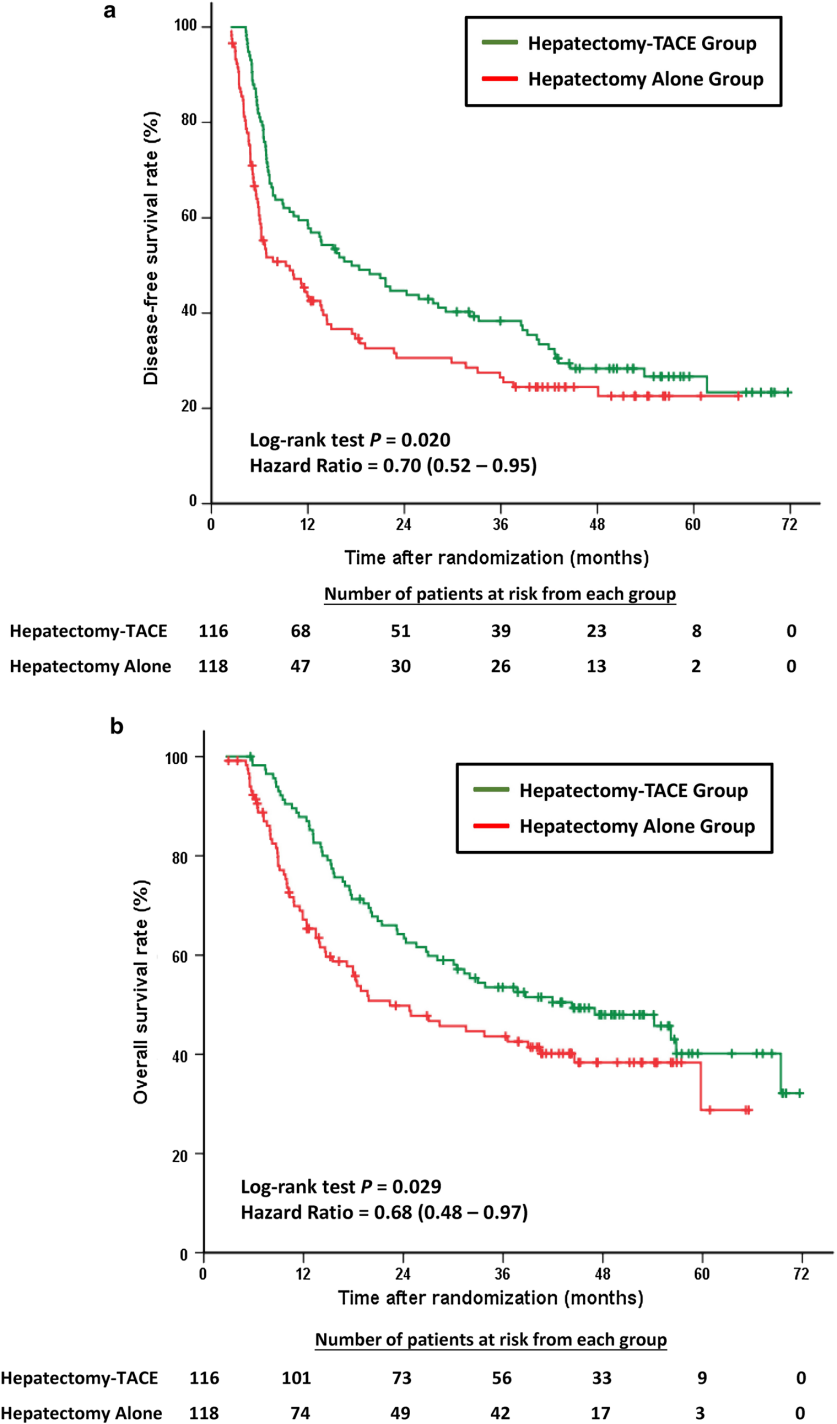
Kaplan-Meier estimates illustrating the differences in **a** disease-free survival (DFS) and **b** overall survival (OS) of the enrolled patients who underwent radical hepatectomy alone against those who had radical hepatectomy and adjuvant TACE. TACE: transcatheter arterial chemoembolization; HR: hazard ratio; CI: confidence interval

The median DFS was 17.45 months (95% CI 11.99–29.14) in the Hepatectomy-TACE group and 9.27 months (95% CI 6.05–13.70) in the Hepatectomy Alone group (HR = 0.70, 95% CI 0.52–0.95, *P* = 0.020; Fig. 2a). By March 31, 2016, 168 (71.8%) of the 234 patients had experienced recurrence (83 in the Hepatectomy-TACE group and 85 in the Hepatectomy Alone group). The 1-, 2-, 3-, and 5-year DFS rates for the Hepatectomy-TACE group were 58.6%, 44.7%, 38.4%, and 26.7% and were 43.5%, 30.6%, 26.5%, and 22.6% for the Hepatectomy Alone group, respectively.

The median OS for the Hepatectomy-TACE group was 44.29 months (95% CI 25.99–62.58) and was 22.37 months (95% CI 10.84–33.91) in the Hepatectomy Alone group (HR = 0.68, 95% CI 0.48–0.97, *P* = 0.029; Fig. 2b). By March 31, 2016, 128 (54.7%) of the 234 enrolled patients had died (62 in the Hepatectomy-TACE group and 66 in the Hepatectomy Alone group). The 1-, 2-, 3-, and 5-year OS rates for the Hepatectomy-TACE group were 87.8%, 64.3%, 53.5%, and 40.2% and were 67.2%, 49.8%, 43.6%, and 28.8% for the Hepatectomy Alone group, respectively.

**Fig. 3:**
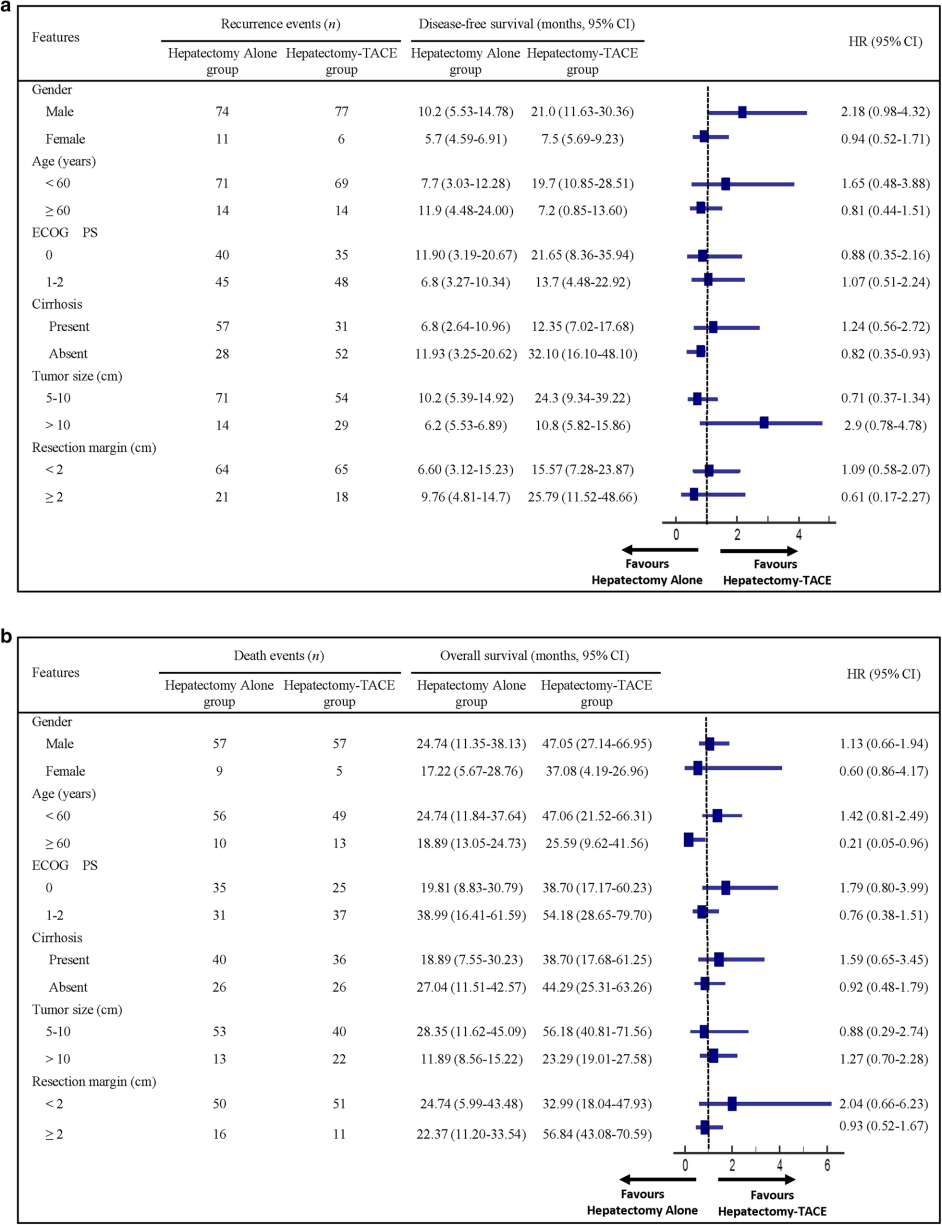
The subgroup analysis of the **a** disease-free survival (DFS) and **b** overall survival (OS) of enrolled patients who underwent radical hepatectomy only compared to those who had radical hepatectomy and adjuvant TACE. HR: hazard ratio; CI: confidence interval; ECOG PS: Eastern Cooperative Oncology Group performance score. Survival data are presented as median with 95% CI in parentheses

The results of subgroup analyses were generally consistent with those of the primary analyses. It indicated that male patients, age < 60 years, presence of cirrhosis, tumor > 10 cm, and resection margin < 2 cm were associated with a greater DFS (Fig. 3a) and OS benefits (Fig. 3b) from adjuvant TACE.

After recurrence, 56 patients (67.5%) in the Hepatectomy-TACE group and 46 patients (54.1%) in the Hepatectomy Alone group underwent subsequent antitumor therapies, including locoregional ablation, hepatectomy, systemic chemotherapy, sorafenib, and TACE (summarized in Table 3).

**Table 3:** The subsequent antitumor therapies prescribed to the enrolled hepatocellular carcinoma patients after diagnosis of tumor recurrence

Antitumor therapy	Hepatectomy-TACE group (*n* = 56)	Hepatectomy Alone group (*n* = 46)	*P*-value
Locoregional ablation	27	8	< 0.001
Hepatectomy^a^	2	2	1.000
Systemic chemotherapy	1	5	0.210
Sorafenib	14	5	0.029
TACE	12	26	0.016

Hepatectomy-TACE: radical hepatectomy followed by adjuvant transcatheter arterial chemoembolization; Hepatectomy Alone: had undergone only radical hepatectomy

^a^ Resection of the recurrent lesion(s) in the liver

### Safety of treatment

Grade 3–4 AEs from the time of randomization to the last day of follow-up were reported in 25 patients (21.6%) from the Hepatectomy-TACE group and 10 patients (8.5%) in the Hepatectomy Alone group (Table 4). Fever, nausea/vomiting, and liver dysfunction were the most common AEs in the Hepatectomy-TACE group. The most common grade 3 or 4 adverse events in both groups were neutropenia and liver dysfunction. However, most AEs were mild and manageable and no toxicity-associated deaths occurred in this study.

**Table 4:** Adverse events of the enrolled patients after hepatectomy from the day of randomization to the last day of follow-up

Adverse events	Hepatectomy-TACE group (*n* = 116)		Hepatectomy Alone group (*n* = 118)		*P*-value
	Grade 1–2	Grade 3–4	Grade 1–2	Grade 3–4	
Neutropenia	16	6	6	2	0.006
Anemia	8	2	4	2	0.312
Thrombocytopenia	15	4	3	1	< 0.001
Fever	29	3	4	0	< 0.001
Pain	16	2	5	1	0.010
Nausea/vomiting	26	1	2	0	< 0.001
Liver dysfunction	39	5	6	3	< 0.001
Fatigue	12	2	4	1	0.032

Hepatectomy-TACE: radical hepatectomy followed by adjuvant transcatheter arterial chemoembolization; Hepatectomy Alone: had undergone only radical hepatectomy

## Discussion

In this open-labeled, randomized, phase III trial, we evaluated the efficacy and safety of adjuvant TACE versus hepatectomy alone among HCC patients with solitary tumor ≥ 5 cm and MVI after curative resection. The results showed that, compared with the Hepatectomy Alone group, the Hepatectomy-TACE group had significantly both prolonged median DFS (17.45 vs. 9.27 months, HR = 0.70, *P* = 0.020) and OS (44.29 vs. 22.37 months, HR = 0.68, *P* = 0.029) from randomization. In subgroup analyses, we found that male patients, age < 60 years, presence of cirrhosis, tumor > 10 cm, and resection margin < 2 cm may derive a greater survival benefit from adjuvant TACE and that these factors should be considered in the selection process for future clinical trials.

MVI is a recognized risk factor for recurrence after hepatectomy in HCC patients. The presence of MVI is associated with multiple factors including tumor size. In an international multicenter study which enrolled 1073 HCC patients, Pawlik et al. [25] reported that the rate of MVI increased with tumor size (≤ 3.0 cm, 25%; 3.1–5.0 cm, 40%; 5.1–6.5 cm, 55%; and > 6.5 cm, 63%) (*P* < 0.005). Among patients with solitary tumor only, MVI occurred more frequently with tumors measuring 5.1–6.5 cm (41%) than for those with tumors measuring ≤ 5.0 cm (27%) (*P* < 0.003). Although wide resection margins may decrease the postoperative recurrence rate and improve survival outcomes [10], however, adequate resection margins were often unachievable due to the cumbersome tumor location and concomitant cirrhosis. Also, MVI beyond the resection margin may become the origin of recurrence. Also, in this study, we excluded patients with solitary tumors < 5 cm because they had a relatively low risk of recurrence due to the low rate of MVI and the high achievability of wide resection margins.

Unfortunately, there is no universally accepted adjuvant therapy for HCC patients with MVI in which efficacy, safety, and cost-effectiveness are conclusive. Some studies have evaluated TACE as a single adjuvant approach or in combination with other therapies (including antiviral therapy and interferon-α) for HCC patients with high risks of recurrence after resection [26–29]. In addition, a recent retrospective study also showed that postoperative adjuvant TACE could prolong the recurrence-free survival (RFS) and OS among HCC patients with MVI [30]. As such, these studies provided the rational evidence to select TACE as an adjuvant therapy in this present study.

Compared with the participants in the above studies, who showed high heterogeneity in tumor stage, the participants were relatively homogeneous in our present study. Solitary HCC is considered as a curable disease, and patients usually undergo more aggressive surgical treatment, although there is no recommended adjuvant therapy in the current official guidelines for these patients with solitary HCC and MVI. To our knowledge, this is the first study to report the value of adjuvant TACE in this specific population.

Interestingly, adjuvant TACE significantly reduced early recurrence rate (within 2 years) after hepatectomy. The 1- and 2-year DFS rates were 58.6% and 44.7% for the Hepatectomy-TACE group and 43.5% and 30.6% for the Hepatectomy Alone group, respectively. However, this difference was less obvious when comparing the 5-year DFS rate between the two groups (26.7% in Hepatectomy-TACE group vs. 22.6% in Hepatectomy Alone group). MVI was found to be the only independent risk factor for early recurrence, which is consistent with our previous study [31]. With the stimulation of multiple growth factors after hepatectomy, occult tumor cells proliferate rapidly and form visible recurrences as the remnant liver regenerates. The high sensitivity of actively proliferating tumor cells to chemotherapeutic agents may be an important reason for the decreased in early recurrence rate in the Hepatectomy-TACE group. Conversely, adjuvant TACE could increase the local concentration of chemotherapeutic agents in the liver, potentially avoiding the undesirable adverse events of systemic chemotherapy.

We also analyzed the underlying reasons for the considerably prolonged OS in the Hepatectomy-TACE group. After diagnosis of tumor recurrence, only 46 patients (54.1%) in the Hepatectomy Alone group underwent subsequent antitumor therapies (such as locoregional ablation, hepatectomy, systemic chemotherapy, sorafenib, and TACE; Table 3), which were less than those in the Hepatectomy-TACE group where greater proportion of patients, 67.5% (56 patients) had antitumor therapies and therefore may have resulted in a shorter OS in the Hepatectomy Alone group. Furthermore, as shown in Table 3, a greater number of patients with recurrence in the Hepatectomy-TACE group underwent locoregional ablation and were prescribed with sorafenib as a subsequent antitumor therapy. This may reflect the fact that recurrence was often localized and controllable. Conversely, more patients with recurrence in the Hepatectomy Alone group underwent relative palliative TACE, which may be associated with more extensive recurrence, as well as unfavorable factors, such as macrovascular tumor thrombus and extrahepatic metastases. However, our results should be interpreted with caution since the impact of adjuvant TACE on recurrence patterns, together with the direct therapeutic effects of adjuvant TACE itself, might collectively contribute to the survival benefits in the Hepatectomy-TACE groups.

As an important trial, the adjuvant sorafenib for hepatocellular carcinoma after resection or ablation (STORM) trial did not reach its primary endpoint of prolonging RFS [19]. The negative results of the STORM trial suggested that antitumor activity against existing or advanced HCC is not necessarily associated with efficacy in the adjuvant setting against micro-metastatic disease. In the absence of established predictive biomarkers of response to sorafenib in patients with advanced HCC, a population potential benefit from adjuvant sorafenib cannot be defined [32]. Besides, the STORM trial underscored the importance of selecting appropriate candidates with a high recurrence risk in such adjuvant settings.

Despite the results of this study demonstrating the superiority of adjuvant TACE over radical hepatectomy alone, there are still some limitations worth mentioning in this study. First, this is a single-center study. To validate the significance of adjuvant TACE in this specific population, a prospective, well-designed, multicenter, and randomized trial is necessary. Second, recent studies have reported that not only the presence of MVI but also the grade of MVI can impact the recurrence and survival of HCC patients [33, 34]. However, we did not investigate the grade of MVI due to the early design of this study protocol. Third, the optimal adjuvant TACE protocol (including chemotherapeutic agents, dosage, and interval) remains to be elucidated and further studies are required.

## Conclusions

Our findings demonstrate the survival and safety benefits of adjuvant TACE in HCC patients with solitary tumor ≥ 5 cm and MVI after curative resection. However, future prospective, multicenter, randomized clinical trials are necessary to evaluate the optimal TACE regimens (including drugs and dosages) and the feasibility of combination with other antitumor therapies.
